# Predictors of myocardial fibrosis and response to anti-fibrotic therapy in heart failure with preserved ejection fraction

**DOI:** 10.1007/s10554-022-02544-9

**Published:** 2022-02-09

**Authors:** Gavin A. Lewis, Anna Rosala-Hallas, Susanna Dodd, Erik B. Schelbert, Simon G. Williams, Colin Cunnington, Theresa McDonagh, Christopher A. Miller

**Affiliations:** 1 Division of Cardiovascular Sciences, School of Medical Sciences, Faculty of Biology, Medicine and Health, Manchester Academic Health Science Centre, University of Manchester, Oxford Road, Manchester, M13 9PL England; 2 Manchester University NHS Foundation Trust, Southmoor Road, Manchester, M23 9LT England; 3 Liverpool Clinical Trials Centre, Clinical Directorate, Faculty of Health and Life Sciences, University of Liverpool (a member of Liverpool Health Partners), Alder Hey Children’s NHS Foundation Trust, Liverpool, L12 2AP England; 4 Department of Health Data Science, Institute of Population Health, Faculty of Health and Life Sciences, University of Liverpool (a member of Liverpool Health Partners), Block F, Waterhouse Bld, 1-5 Brownlow Street, Liverpool, L69 3GL England; 5 Department of Medicine, University of Pittsburgh School of Medicine, Pittsburgh, PA USA; 6 UPMC Cardiovascular Magnetic Resonance Center, Heart and Vascular Institute, Pittsburgh, PA USA; 7 Clinical and Translational Science Institute, University of Pittsburgh, Pittsburgh, PA USA; 8 King’s College Hospital, Denmark Hill, London, SE5 9RS UK; 9 Division of Cell-Matrix Biology & Regenerative Medicine, School of Biology, Faculty of Biology, Medicine & Health, Manchester Academic Health Science Centre, Wellcome Centre for Cell-Matrix Research, University of Manchester, Oxford Road, Manchester, M13 9PT England

**Keywords:** Heart failure, Myocardial fibrosis, Magnetic resonance imaging (MRI), Extracellular volume (ECV)

## Abstract

Myocardial fibrosis, measured using magnetic resonance extracellular volume (ECV), associates with adverse outcome in heart failure with preserved ejection fraction (HFpEF). In the PIROUETTE (The Pirfenidone in Patients with Heart Failure and Preserved Left Ventricular Ejection Fraction) trial, the novel anti-fibrotic agent pirfenidone reduced myocardial fibrosis. We sought to identify baseline characteristics that associate with myocardial fibrotic burden, the change in myocardial fibrosis over a year, and predict response to pirfenidone in patients with HFpEF. Amongst patients enrolled in the PIROUETTE trial (n = 107), linear regression models were used to assess the relationship between baseline variables and baseline myocardial ECV, with change in myocardial ECV adjusting for treatment allocation, and to identify variables that modified the pirfenidone treatment effect. Body mass index, left atrial reservoir strain, haemoglobin and aortic distensibility were associated with baseline ECV in stepwise modelling, and systolic blood pressure, and log N-terminal pro B-type natriuretic peptide were associated with baseline ECV in clinically-guided modelling. QRS duration, left ventricular mass and presence of an infarct at baseline were associated with an increase in ECV from baseline to week 52. Whilst QRS duration, presence of an infarct, global longitudinal strain and left atrial strain modified the treatment effect of pirfenidone when considered individually, no variable modified treatment effect on multivariable modelling. Baseline characteristics were identified that associate with myocardial fibrosis and predict change in myocardial fibrosis. No variables that independently modify the treatment effect of pirfenidone were identified (PIROUETTE, NCT02932566).

**Supplementary Information:**

The online version contains supplementary material available at 10.1007/s10554-022-02544-9.

## Introduction

Myocardial fibrosis, measured using cardiovascular magnetic resonance (CMR) extracellular matrix volume (ECV), is associated with adverse outcome in patients with heart failure with preserved ejection fraction (HFpEF), including hospitalisation for heart failure (HF) and death [1–5].

The Pirfenidone in patients with heart failure and preserved left ventricular ejection fraction (PIROUETTE) study was a phase II, double-blind, placebo-controlled, randomised trial designed to evaluate the efficacy and mechanism of the novel anti-fibrotic agent, pirfenidone, in patients with HFpEF and myocardial fibrosis [6]. Pirfenidone is an orally bioavailable, small molecule anti-fibrotic agent that inhibits cardiac fibroblast synthesis and secretion of TGF-β1, proliferation and activation of fibroblasts, and profibrotic pathways. In the trial, treatment with pirfenidone for 52 weeks reduced myocardial fibrosis.

Identification of characteristics that are associated with myocardial fibrosis at baseline, and which predict change in myocardial fibrosis over time and response to pirfenidone, would improve risk stratification of patients with HFpEF, guide patient management, and guide future trial recruitment.

This analysis of data from the PIROUETTE trial aimed to identify baseline characteristics that associate with baseline myocardial fibrotic burden, predict change in myocardial fibrosis over one year, and predict response pirfenidone, in patients with HFpEF.

## Methods

### Study design and patient selection

Between March 7, 2017, to December 19, 2018, the PIROUETTE trial (Clinicaltrials.gov NCT02932566) randomised 94 patients with HFpEF and myocardial fibrosis to pirfenidone or placebo for 52-weeks. The trial design and results have previously been published [6, 7]. Eligibility requirements included patients ≥ 40 years of age, symptoms and signs of heart failure, left ventricular ejection fraction of ≥ 45%, and elevated natriuretic peptides (brain natriuretic peptide (BNP) ≥ 100 pg/ml or N-terminal pro-B-type natriuretic peptide (NT-proBNP) ≥ 300 pg/ml; or BNP ≥ 300 pg/ml or NT-proBNP ≥ 900 pg/ml if atrial fibrillation (AF) present). Eligible patients underwent CMR and those with evidence of myocardial fibrosis, defined as an ECV of 27% or higher, were randomised in a 1:1 ratio to treatment with either pirfenidone or matching placebo for 52 weeks using block randomisation, stratified by sex. Those without myocardial fibrosis were entered into a registry (n = 13). Key exclusion criteria included alternative causes of patients’ symptoms such as significant pulmonary disease, anaemia, or obesity; pericardial constriction, hypertrophic cardiomyopathy or infiltrative cardiomyopathy; and contraindication to magnetic resonance imaging. The primary outcome was change in myocardial fibrosis, measured using CMR ECV, from baseline to 52 weeks.

The trial was sponsored by Manchester University NHS Foundation Trust. Trial management, independent data management and independent statistical analyses were performed by Liverpool Clinical Trials Centre, a United Kingdom Clinical Research Collaboration fully-registered Clinical Trials Unit. The study protocol was approved by a research ethics committee and trial conduct was overseen by a trial steering committee. Patients were identified at six UK hospitals. Study visits took place at Manchester University NHS Foundation Trust. All patients provided written informed consent.

### Study procedures

The protocol, trial procedures, analysis methods and outcome measurements have been described previously [6, 7]. In brief, CMR, echocardiography, electrocardiography, 6 min walk test, laboratory tests and the Kansas City Cardiomyopathy Questionnaire (KCCQ) were performed at baseline and repeated after 52 weeks of treatment.

Myocardial ECV was calculated from basal and mid left ventricular (LV) short axis T1 maps (MOdified Look-Locker Inversion recovery [MOLLI]), acquired before and 15 min following gadolinium contrast (0.15 mmol/kg of gadoterate meglumine), as: ECV = (1–haematocrit) x [ΔR1_myocardium_] / [ΔR1_bloodpool_], where ΔR1 is the difference in relaxation rates (1/T1) between pre- and post-contrast [4]. Haematocrit was measured on the same day as CMR scanning. Absolute myocardial extracellular matrix (ECM) volume was calculated as the product of LV myocardial volume (LV mass divided by the specific gravity of myocardium [1.05 g/ml]) and ECV. Absolute myocardial cellular volume was calculated as the product of LV myocardial volume (LV mass divided by the specific gravity of myocardium [1.05 g/ml]) and (1–ECV). Further details can be found in the trial protocol paper [7].

### Statistical analysis

The trial was analysed and reported according to the ‘Consolidated Standard of Reporting Trials’ (CONSORT) and the International Conference on Harmonisation E9 guidelines. Analysis was conducted on an intention to treat basis, including all randomised patients retained in their randomised treatment groups. Continuous data are presented as mean ± standard deviation (SD) or as median (interquartile range (IQR)), as appropriate. Categorical data are presented as counts and percentages.

Univariable and multivariable linear regression models were used to assess the relationship between baseline variables and baseline myocardial ECV. Variablesfor which p-values were < 0.3 in the univariable analyses were included in forward stepwise selection models, with p-value thresholds of 0.05 for entry and 0.1 for removal. The chosen variables were then included in multivariable regression models, where the outcome variable was baseline myocardial ECV. Collinearity was investigated by assessing correlation between variables and by assessing the variance impact factor for each model, which confirmed that collinearity correction measures were not required. Additional models using absolute myocardial ECM volume and absolute myocardial cell volume as outcome variables were constructed to assess ECV component associations. ‘Clinically-guided’ multivariable models were also constructed that included variables that, based on the published literature and clinical judgment, were hypothesised to be associated with myocardial ECV. The variables included in the ‘clinically-guided’ models were limited to 10 variables: age, sex, body mass index, diabetes, atrial fibrillation, log-N-terminal pro B-type natriuretic peptide (NT-proBNP), left atrial strain, systolic blood pressure, right ventricular end diastolic volume index and global longitudinal strain.

Analysis of variance (ANOVA) models were used to assess the relationship between baseline variables and change in myocardial ECV (week 52 value minus baseline value), adjusting for treatment allocation. Variables for which p-values were < 0.3 in the ANOVA models were included in forward stepwise selection models, with p-value thresholds of 0.05 for entry and 0.1 for removal. Treatment allocation was forced into the selection model. The chosen variables were then included in a multivariable regression model, where the outcome variable was change in myocardial ECV (week 52 value minus baseline value). Additional models using absolute myocardial ECM volume and absolute myocardial cell volume as outcome variables were constructed to assess ECV component associations. ‘Clinically-guided’ multivariable models, as described above, were also constructed, limited to eight variables: age, sex, diabetes, log NT-proBNP, left atrial strain (reservoir), systolic blood pressure, right ventricular end diastolic volume index and global longitudinal strain.

In order to provide an indication of which baseline variables predicted a response to pirfenidone, linear regression was used to model week 52 myocardial ECV with each baseline variable in turn, including an interaction between treatment allocation and each baseline variable, adjusting for baseline myocardial ECV, in order to identify baseline variables that modified treatment effect. Variables identified as being treatment effect modifiers were subsequently included in a multivariable ordinary least squares regression model, where the outcome was week 52 myocardial ECV. All analyses were performed in SAS (Version 9.4, SAS Institute, Inc.; Cary, NC).

## Results

### Patients

**Fig. 1:**
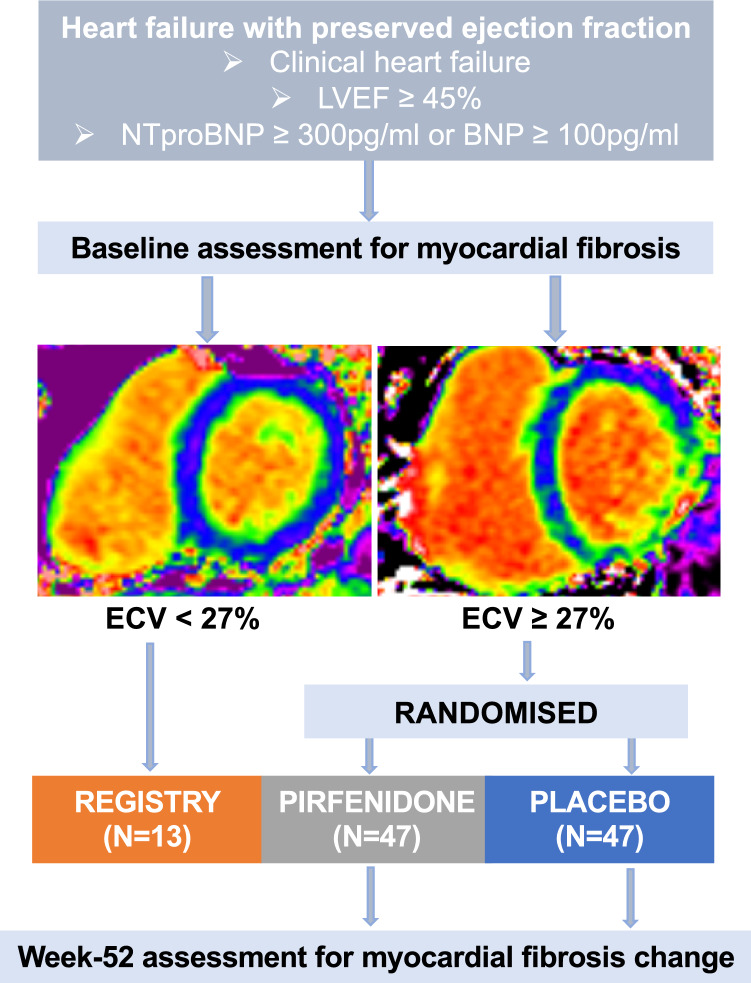
Design of the PIROUETTE trial. Extracellular matrix volume (ECV) quantifies the extent of myocardial fibrosis. ECV maps are shown with normal myocardium (ECV 24.9%) and myocardial fibrosis (ECV 31.3%). Measurements were repeated after 52 weeks of treatment with pirfenidone or placebo. BNP brain natriuretic peptide; LVEF left ventricular ejection fraction; NTproBNP N-terminal pro-B-type natriuretic peptide

107 patients were enrolled, including 94 who had evidence of myocardial fibrosis (ECV ≥ 27%) who were randomised, and 13 patients without evidence of myocardial fibrosis (ECV < 27%) who were not randomised (Fig. 1). Baseline characteristics are summarised in Table 1. Mean age of patients was 77 years, and 49% were female. Nearly all patients had New York Heart Association functional class II or III symptoms (94%), mean left ventricular ejection fraction was 65% and median NT-proBNP was 1067 pg/ml. At the end of the trial, 12 patients had withdrawn from the study and two had died. No patient was lost to follow-up. A total of 80 patients were therefore included in the analyses evaluating change in ECV and pirfenidone treatment response.

**Table 1:** Baseline characteristics

Characteristics	N = 107
Age—yrs	77 ± 8
Female—no. (%)	52(49)
Hypertension—no. (%)	89(83)
Diabetes—no. (%)	30(28)
Atrial fibrillation—no. (%)	48(45)
Current or ex-smoker—no. (%)	41(38)
NYHA Class—no. (%)	
I	6(6)
II	56(52)
III	45(42)
Systolic blood pressure—mmHg	138 ± 23
Diastolic blood pressure—mmHg	76 ± 15
BMI—kg/m^2^	31 ± 6
eGFR—ml/min	57 ± 17
Haemoglobin—g/dL	13.0 ± 1.5
Median NT-proBNP—pg/ml (IQR)	1067(493–2152)
HS-Troponin T—pg/ml	26.3 ± 29.5
QRS duration—ms	106 ± 20
Myocardial ECV—%	29.4 ± 3.1
Absolute myocardial ECM volume—ml	36.1 ± 11.2
Absolute myocardial cell volume—ml	86.6 ± 24.6
Infarct LGE—no. (%)	31(29)
LV end diastolic volume index—ml/m^2^	63 ± 18
LV ejection fraction—%	65 ± 16
LV mass index—g/m^2^	65 ± 15
Average e’—cm/s	8.7 ± 2.4
Average E/e’—cm/s	12.2 ± 3.4
Global longitudinal strain—%	− 16.1 ± 3.6
Torsion—degrees/cm	1.5 ± 0.7
RV end diastolic volume index—ml/m^2^	69 ± 16
RV ejection fraction—%	53 ± 10
Pulmonary artery systolic pressure—mmHg	33 ± 13
LA volume index—ml/m^2^	70 ± 18
LA strain (reservoir)—%	17.6 ± 8.0
LA strain (booster)—%	12.7 ± 4.3
LA strain (conduit)—%	10.5 ± 3.9
Aortic distensibility—10^–3^/mmHg	1.5 ± 0.9
Pulse Wave Velocity—m/s	12.6 ± 5.0
6-min walk test—m	269 ± 115
KCCQ Clinical Summary Score	56 ± 20

Values are means ± SD unless specified

*BMI* body mass index, *ECM* extracellular matrix, *ECV* extracellular matrix volume, *eGFR* estimated glomerular filtration rate, *HS-Troponin T* high-sensitivity troponin T, *IQR* interquartile range, *KCCQ* Kansas city cardiomyopathy questionnaire, *LA* left atrial, *LGE* late gadolinium enhancement, *LV* left ventricular, *NT-proBNP* N-terminal pro B-type natriuretic peptide, *NYHA* New York Heart Association, *RV* right ventricular

### Associations with baseline myocardial fibrosis

In the stepwise multivariable analysis, lower body mass index, left atrial strain (reservoir), haemoglobin and higher aortic distensibility were associated with higher ECV at baseline (Table 2). There was also a strong trend towards an association between log NT-proBNP and ECV. In the ‘clinically-guided’ multivariable model, lower body mass index and systolic blood pressure, and higher log NT-proBNP, were associated with higher ECV at baseline, and there was a strong trend towards an association between lower global longitudinal strain and ECV.

**Table 2:** Associations between selected patient characteristics and myocardial extracellular volume (ECV) at baseline

Baseline covariate	Forward stepwise selection model						Clinical multivariable model		
	Univariable model			Multivariable model					
	β-coefficient (SE)	95% CI	P-value	β-coefficient (SE)	95% CI	P-value	β-coefficient (SE)	95% CI	P-value
Age—yrs	0.04(0.04)	− 0.04–0.12	0.32				− 0.03(0.04)	− 0.11–0.06	0.54
Gender (Female vs Male)	− 1.13(0.60)	− 2.32–0.05	0.06				− 0.38(0.62)	− 1.60–0.84	0.54
Atrial fibrillation (Yes vs No)	1.67(0.59)	0.50–2.83	0.006				− 0.40(0.91)	− 2.22–1.41	0.66
Diabetes (Yes vs No)	0.08(0.68)	− 1.26–1.42	0.91				− 0.02(0.66)	− 1.32–1.29	0.98
Current or ex-smoker (Yes vs No)	− 0.71(0.62)	− 1.94–0.52	0.26						
Systolic blood pressure—mmHg	− 0.04(0.01)	− 0.07 to − 0.02	< 0.001				− 0.03(0.01)	− 0.06 to − 0.00	0.03
Diastolic blood pressure—mmHg	− 0.07(0.02)	− 0.11 to − 0.03	< 0.001						
BMI—kg/m^2^	− 0.18(0.05)	− 0.28 to − 0.08	< 0.001	− 0.19(0.05)	− 0.29 to − 0.10	< 0.001	− 0.14(0.05)	− 0.25 to − 0.04	0.009
Haemoglobin—g/dL	− 0.49(0.20)	− 0.89 to − 0.09	0.02	− 0.69(0.18)	− 1.05 to − 0.34	< 0.001			
Log-NT-proBNP—pg/ml	1.39(0.29)	0.82–1.97	< 0.001	0.62(0.36)	− 0.09–1.33	0.09	0.83(0.41)	0.02–1.64	0.045
HS Troponin T—pg/ml	0.03(0.01)	0.01–0.05	0.003						
Infarct LGE (Yes vs No)	1.31(0.66)	0.01–2.61	0.05						
LV mass index—g/m^2^	0.03(0.02)	− 0.01–0.07	0.20						
Global longitudinal strain—%	0.03(0.09)	− 0.14–0.20	0.72				− 0.17(0.09)	− 0.35–0.02	0.07
RV end diastolic volume index—ml/m^2^	0.01(0.02)	− 0.03–0.04	0.75				0.02(0.02)	− 0.02–0.05	0.42
LA volume index—ml/m^2^	0.05(0.02)	0.02–0.08	0.003						
LA strain (reservoir)—%	− 0.13(0.04)	− 0.20 to − 0.06	< 0.001	− 0.11(0.04)	− 0.20 to − 0.03	0.01	− 0.09(0.06)	− 0.21–0.03	0.12
LA strain (booster)—%	− 0.23(0.08)	− 0.39 to − 0.06	0.008						
Aortic distensibility—10^–3^/mmHg	0.43(0.35)	− 0.27–1.13	0.22	0.69(0.29)	0.11–1.27	0.02			

Patients with complete covariate data were included in analyses (n = 104). Variables for which P-value < 0.3 in univariable regression model included within stepwise forward selection multivariable regression model

*BMI* body mass index, *CI* confidence interval, *HS-Troponin T* high-sensitivity troponin T, *LA* left atrial, *LGE* late gadolinium enhancement, *LV* left ventricular, *NT-proBNP* N-terminal pro B-type natriuretic peptide, *RV* right ventricular, *SE* standard error (see Table S1 in Supplementary Appendix for full univariable analyses)

Multivariable models for extracellular and cellular myocardial components revealed that the associations between log NT-proBNP and impaired left atrial strain (reservoir), and baseline ECV, were predominantly driven by associations with baseline myocardial ECM volume (Supplementary Tables S2-3). The inverse associations between body mass index, systolic blood pressure, global longitudinal strain, and baseline ECV, were predominantly driven by positive associations with baseline myocardial cell volume (Supplementary Tables S4-5).

### Predictors of change in myocardial fibrosis

In stepwise multivariable analysis, shorter baseline QRS duration, higher baseline LV mass and presence of an infarct at baseline were associated with an increase in change in ECV from baseline to week 52. In the ‘clinically-guided’ multivariable model, only impaired baseline global longitudinal strain was associated with an increase in change in ECV (Table 3).

**Table 3:** Associations between baseline characteristics and change in myocardial extracellular volume (ECV) from baseline to 52 weeks

Baseline covariate	Forward stepwise selection model						Clinical multivariable model		
	Univariable model			Multivariable model					
	β-coefficient (SE)	95% CI	P-value	β-coefficient (SE)	95% CI	P-value	β-coefficient (SE)	95% CI	P-value
Age—yrs	− 0.00(0.03)	− 0.07–0.06	0.96				0.03(0.04)	− 0.05–0.10	0.51
Gender (Female vs Male)	− 0.68(0.44)	− 1.56–0.20	0.13				− 0.28(0.47)	− 1.23–0.66	0.55
Diabetes (Yes vs No)	0.85(0.51)	− 0.17–1.87	0.10				1.05(0.59)	− 0.12–2.22	0.08
Atrial fibrillation (Yes vs No)	0.51(0.45)	− 0.38–1.40	0.25						
Current or ex-smoker (Yes vs No)	0.73(0.47)	− 0.21–1.67	0.13						
Systolic blood pressure—mmHg	− 0.01(0.01)	− 0.03–0.01	0.49				0.00(0.01)	− 0.02–0.02	0.98
Diastolic blood pressure—mmHg	0.02(0.02)	− 0.01 –0.05	0.21						
Log-NT-proBNP—pg/ml	0.38(0.25)	− 0.10–0.87	0.12				0.17(0.33)	− 0.48–0.83	0.60
HS Troponin T—pg/ml	0.03(0.01)	− 0.00–0.05	0.08						
QRS duration – ms	− 0.03(0.01)	− 0.05 to − 0.00	0.04	− 0.03(0.01)	− 0.05 to − 0.00	0.02			
Infarct LGE (Yes vs No)	1.20(0.45)	0.30 to 2.10	0.009	1.13(0.43)	0.27 to 2.00	0.01			
LV ejection fraction—%	− 0.04(0.03)	− 0.09–0.02	0.18						
LV mass index—g/m^2^	0.03(0.01)	− 0.00–0.05	0.07	0.03(0.01)	0.00–0.05	0.05			
Global longitudinal strain—%	0.14(0.06)	0.02–0.26	0.02				0.17(0.08)	0.01–0.33	0.04
RV end diastolic volume index—ml/m^2^	− 0.00(0.01)	− 0.03–0.03	0.84				0.00(0.02)	− 0.03–0.03	0.88
LA volume index—ml/m^2^	0.01(0.01)	− 0.01–0.04	0.22						
LA strain (reservoir)—%	− 0.05(0.03)	− 0.11–0.01	0.10				0.00(0.04)	− 0.08–0.08	0.95
Pulse Wave Velocity—m/s	0.06(0.05)	− 0.03–0.15	0.23						

All patients who completed the study were included in the stepwise selection model (n = 80). The clinical model included all patients with complete covariate data (n = 79). Variables for which P-value < 0.3 in univariable regression model included within stepwise forward selection multivariable regression model, adjusted for treatment allocation. *CI* confidence interval, *HS-Troponin T* high-sensitivity troponin T, *LA* left atrial, *LGE* late gadolinium enhancement, *LV* left ventricular, *NT-proBNP* N-terminal pro B-type natriuretic peptide, *RV* right ventricular, *SE* standard error (see Table S6 in Supplementary Appendix for full univariable analyses)

Multivariable models for cellular and extracellular myocardial components revealed that a diagnosis of diabetes at baseline was associated with an increase in change in myocardial ECM volume from baseline to week 52, and lower baseline renal function and diastolic blood pressure were associated with an increase in change in myocardial cellular volume from baseline to week 52 (Supplementary Tables S7-10).

### Predictors of response to pirfenidone

Shorter QRS duration, the presence of an infarct, impaired global longitudinal strain and impaired left atrial strain (conduit) were each associated with a greater treatment effect of pirfenidone on 52 week ECV when considered individually. However, when these variables were entered into the same model, no baseline variable independently modified the treatment effect of pirfenidone (Supplementary Figure S1 and Tables S11-12).

## Discussion

This analysis of data from the PIROUETTE trial identified several baseline characteristics that associated with myocardial fibrotic burden. Metrics reflecting important processes in the pathophysiology of HFpEF that associated with myocardial fibrosis included abnormal atrial mechanical remodelling and adaptation to elevated left ventricular filling pressures via natriuretic peptide release. Characteristics that predicted change in myocardial fibrosis over one year included those reflecting a more advanced stage of abnormal LV remodelling at baseline (e.g., previous infarct and increased LV mass). Baseline QRS duration, presence of an infarct, global longitudinal strain and left atrial strain modified the treatment effect of pirfenidone when considered individually, but no baseline variable modified treatment effect on multivariable modelling.

Parametric mapping techniques (T1, T2, T2*, ECV) are now standard sequences utilised in clinical CMR that provide non-invasive assessment of myocardial tissue composition and characterisation. ECV measurement not only identifies extracellular matrix infiltration and expansion, as seen in cardiac amyloidosis and myocardial fibrosis, but can reliably quantify myocardial fibrotic burden with high reproducibility, thereby allowing serial tracking and assessment of response to anti-fibrotic therapy.

The baseline characteristics found to associate with baseline myocardial fibrosis in HFpEF in the current study are in keeping with the study by Kanagala et al. [8], which similarly demonstrated body mass index, haemoglobin and natriuretic peptides to be associated with ECV. Kanagala et al. also found left atrial volume index to be associated with ECV, whereas in the current study, left atrial volume index was outperformed by left atrial strain (reservoir). Measurements of left atrial strain correlate with left atrial myocardial fibrosis assessed using CMR late gadolinium enhancement, and may associate with adverse outcome in HFpEF more strongly than left atrial volume index [9, 10]. In the main analysis of the PIROUETTE trial, treatment with pirfenidone for 52 weeks did not lead to changes in left atrial strain or left atrial volume in comparison to placebo, although the trial was not powered for secondary outcomes. The origins of left atrial dysfunction in HFpEF and its potential role as a distinct disease mechanism, and the relationship between ventricular and atrial fibrosis, remain unclear and require further research [11].

An association was identified between aortic distensibility and myocardial fibrosis. Aortic distensibility measures the change in the cross-sectional area of the ascending aorta (measured at the level of the main pulmonary artery) corrected for pulse pressure and therefore reflects aortic compliance. As compliance and stiffness are reciprocals it is commonly used as an inverse metric of aortic stiffness i.e., distensibility reduces as stiffness increases. Increased central arterial stiffness and magnitude of arterial wave reflections are well recognised in subgroups of patients with HFpEF [12–14], however the relationship between myocardial fibrosis and aortic stiffness is less clear. Interestingly, when patients with HFpEF subjected to invasive haemodynamic assessment were dichotomized according to median ECV in a study by Rommel et al. [15], both groups showed a pathological upward shift of the end-diastolic pressure–volume relationship during exercise; however, the dominant pathophysiology was an increase in myocardial passive stiffness in patients with elevated ECV, whereas increased arterial elastance was a dominant mechanism in patients with a below-median ECV. Both groups demonstrated similar echocardiographic diastolic parameters. Similarly, in a study of patients with hypertensive heart disease, patients with concentric LV remodelling demonstrated the lowest ECV and lowest aortic distensibility [16]. Furthermore, in a preclinical model investigating HFpEF using a diabetic obesity model, aortic fibrosis and reduced aortic distensibility both occurred without any significant myocardial fibrosis being induced, and the measured increase in myocardial stiffness was related to titin isoform shift and not myocardial fibrosis [17]. This would suggest independent pro-fibrotic disease pathways, however in the main analysis of the PIROUETTE trial, treatment with pirfenidone for 52 weeks was associated with a trend towards an improvement in aortic distensibility in comparison to placebo but the difference was not statistically significant (p = 0.078). Whilst it is a well-recognised phenomena and mechanistic phenotype in HFpEF the cellular and pathological determinants of aortic stiffness in HFpEF are poorly understood. Several hypotheses could be postulated relating to the activation of differing pro-fibrotic disease pathways within the myocardium and/or arterial wall leading to arterial stiffness that may be equally receptive to the effects of pirfenidone, however further investigation to define these is required.

The current study is the first to investigate characteristics that predict change in myocardial fibrosis over time in HFpEF. Given the association between myocardial fibrosis and adverse outcome in HFpEF, identification of factors that predict an increase in myocardial fibrotic burden may help to risk stratify patients. Remote myocardial fibrosis is well recognised post-myocardial infarction and, in studies using ECV, remote myocardial fibrosis is predictive of adverse remodelling post-infarction, although these studies have focused on the early period (first 3–6 months) post-infarction [18, 19]. It is interesting that the current study indicates that the presence of an infarct is associated with progression of remote myocardial fibrosis; possibly identifying a subset of patients with HFpEF in whom myocardial fibrosis is a key disease mechanism, who potentially, therefore, may be more likely to derive benefit from antifibrotic intervention. In the PARAGON-HF trial of angiotensin–neprilysin inhibition in heart failure with preserved ejection fraction, subgroup analysis suggested participants with a lower left ventricular ejection fraction may derive benefit from the intervention. Patients with a previous myocardial infarction could be expected to have a lower ejection fraction, and it is possible that the current findings may provide some mechanistic insight into this subgroup finding, but this hypothesis requires investigation. No association was found between blood pressure and change in ECV in both univariable and clinically-guided multivariable models.

Regarding the identified association between baseline global longitudinal strain and change in ECV, as discussed earlier, the relationship between myocardial ECV and myocardial mechanics is not straightforward, and, whilst some cross sectional data have shown associations between ECV and global longitudinal strain, in the largest cohort to date (albeit in unselected patients undergoing clinically-indicated CMR rather than specifically patients with HF), cross-sectional myocardial ECV correlated minimally with global longitudinal strain (R^2^ = 0.04). Given the multiple statistical testing employed, the findings of the current study require confirmation in larger natural history studies.

No baseline variable was identified to modify the treatment effect of pirfenidone on multivariable modelling. This may be because no baseline variables independently associate with the pirfenidone treatment effect, or it may be that the study did not have enough power to identify any such variables. The finding that the presence of an infarct was associated with a greater treatment effect of pirfenidone, when variables were assessed individually, may suggest patients with HFpEF who have had an infarct may derive benefit from antifibrotic intervention as discussed earlier, but the finding did not hold on multivariable analysis and further investigation is required.

## Limitation

The sample size for the PIROUETTE study was calculated based on the primary outcome. The trial was not powered for secondary outcomes, or the multiple statistical tests performed in this study, thus the findings of this study are considered exploratory. The analyses conducted as part of the current study were not included in the Statistical Analysis Plan for PIROUETTE and thus are considered post-hoc. Nevertheless, the analyses conducted in this study were prespecified in an ‘Additional Statistical Analysis Plan’ that was written before trial data lock.

## Conclusion

In this analysis of data from the PIROUETTE trial, baseline characteristics reflecting atrial mechanical remodelling and elevated left ventricular filling pressures were associated with baseline myocardial fibrotic burden. Fibrotic progression over one year was associated with characteristics reflecting abnormal LV remodelling at baseline, however no baseline variables were identified that independently modified the treatment effect of pirfenidone in HFpEF.

### Supplementary Information

Below is the link to the electronic supplementary material.

Supplementary file1 (PDF 613 KB)

## Abbreviations

AF: Atrial fibrillation
ANCOVA: Analysis of covariance
BMI: Body mass index
BNP: Brain natriuretic peptide
CMR: Cardiac magnetic resonance
ECM: Extracellular matrix
ECV: Extracellular matrix volume
HFpEF: Heart failure with preserved ejection fraction
NT-proBNP: N-terminal pro B-type natriuretic peptide

## References

[1] Fontana M, White SK, Banypersad SM (2012). Comparison of T1 mapping techniques for ECV quantification. Histological validation and reproducibility of ShMOLLI versus multibreath-hold T1 quantification equilibrium contrast CMR. J Cardiovasc Magn Reson.

[2] Miller CA, Naish JH, Bishop P (2013). Comprehensive validation of cardiovascular magnetic resonance techniques for the assessment of myocardial extracellular volume. Circ Cardiovasc Imaging.

[3] Schelbert EB, Fridman Y, Wong TC (2017). Temporal relation between myocardial fibrosis and heart failure with preserved ejection fraction: association with baseline disease severity and subsequent outcome. JAMA Cardiol.

[4] Messroghli DR, Moon JC, Ferreira VM (2017). Clinical recommendations for cardiovascular magnetic resonance mapping of T1, T2, T2* and extracellular volume: a consensus statement by the society for cardiovascular magnetic resonance (SCMR) endorsed by the European Association for Cardiovascular Imaging (EACVI). J Cardiovasc Magn Reson.

[5] Roy C, Slimani A, de Meester C (2018). Associations and prognostic significance of diffuse myocardial fibrosis by cardiovascular magnetic resonance in heart failure with preserved ejection fraction. J Cardiovasc Magn Reson.

[6] Lewis GA, Dodd S, Clayton D (2021). Pirfenidone in heart failure with preserved ejection fraction: a randomized phase 2 trial. Nat Med.

[7] Lewis GA, Schelbert EB, Naish JH (2019). Pirfenidone in heart failure with preserved ejection fraction-rationale and design of the pirouette trial. Cardiovasc Drugs Ther.

[8] Kanagala P, Cheng ASH, Singh A (2019). Relationship between focal and diffuse fibrosis assessed by CMR and clinical outcomes in heart failure with preserved ejection fraction. JACC Cardiovasc Imaging.

[9] Freed BH, Daruwalla V, Cheng JY (2016). Prognostic utility and clinical significance of cardiac mechanics in heart failure with preserved ejection fraction: importance of left atrial strain. Circ Cardiovasc Imaging.

[10] Kuppahally SS, Akoum N, Burgon NS (2010). Left atrial strain and strain rate in patients with paroxysmal and persistent atrial fibrillation: relationship to left atrial structural remodeling detected by delayed-enhancement MRI. Circ Cardiovasc Imaging.

[11] Santos AB, Roca GQ, Claggett B (2016). Prognostic relevance of left atrial dysfunction in heart failure with preserved ejection fraction. Circ Heart Fail.

[12] Mohammed SF, Borlaug BA, Roger VL (2012). Comorbidity and ventricular and vascular structure and function in heart failure with preserved ejection fraction: a community-based study. Circ Heart Fail.

[13] Reddy YNV, Andersen MJ, Obokata M (2017). Arterial stiffening with exercise in patients with heart failure and preserved ejection fraction. J Am Coll Cardiol.

[14] Weber T, Wassertheurer S, O'Rourke MF (2013). Pulsatile hemodynamics in patients with exertional dyspnea: potentially of value in the diagnostic evaluation of suspected heart failure with preserved ejection fraction. J Am Coll Cardiol.

[15] Rommel KP, von Roeder M, Latuscynski K (2016). Extracellular volume fraction for characterization of patients with heart failure and preserved ejection fraction. J Am Coll Cardiol.

[16] Rodrigues JC, Amadu AM, Dastidar AG (2016). Comprehensive characterisation of hypertensive heart disease left ventricular phenotypes. Heart.

[17] Reil JC, Hohl M, Reil GH (2013). Heart rate reduction by If-inhibition improves vascular stiffness and left ventricular systolic and diastolic function in a mouse model of heart failure with preserved ejection fraction. Eur Heart J.

[18] Bulluck H, Rosmini S, Abdel-Gadir A (2016). Automated extracellular volume fraction mapping provides insights into the pathophysiology of left ventricular remodeling post-reperfused st-elevation myocardial infarction. J Am Heart Assoc.

[19] Carberry J, Carrick D, Haig C (2016). Remote zone extracellular volume and left ventricular remodeling in survivors of st-elevation myocardial infarction. Hypertension.

