# Supplementary material for: Predictors of myocardial fibrosis and response to anti-fibrotic therapy in heart failure with preserved ejection fraction
Source: Int J Cardiovasc Imaging. 2022 Feb 9;38(7):1569–78. doi: 10.1007/s10554-022-02544-9 (PMC9797453; doi:10.1007/s10554-022-02544-9)
Supplement: Supplementary file 1 — Supplementary file1 (PDF 613 KB) [file 10554_2022_2544_MOESM1_ESM.pdf]

Predictors of myocardial fibrosis and response to anti-fibrotic therapy in heart failure with preserved ejection fraction

SUPPEMENTARY APPENDIX

Table of Contents

SUPPLEMENTARY TABLES 2

Supplementary Table S1. Association between patient characteristics and myocardial extracellular volume (ECV) at baseline 2

Supplementary Table S2. Association between patient characteristics and absolute myocardial extracellular matrix (ECM) volume at baseline 4

Supplementary Table S3. Association between patient characteristics and absolute myocardial extracellular matrix (ECM) volume at baseline (clinical model) 6

Supplementary Table S4. Association between patient characteristics and absolute myocardial cell volume at baseline. 7

Supplementary Table S5. Association between patient characteristics and absolute myocardial cell volume at baseline (clinical model) 9

Supplementary Table S6. Associations between baseline characteristics and change in myocardial extracellular volume (ECV) from baseline to 52 weeks 10

Supplementary Table S7. Association between patient characteristics and change in absolute myocardial extracellular matrix (ECM) volume from baseline to 52 weeks 12

Supplementary Table S8. Association between patient characteristics and change in absolute myocardial extracellular matrix (ECM) volume from baseline to 52 weeks (clinical model) 14

Supplementary Table S9. Association between patient characteristics and change in absolute myocardial cell volume from baseline to 52 weeks 15

Supplementary Table S10. Association between patient characteristics and change in absolute myocardial cell volume from baseline to 52 weeks (clinical model) 17

Supplementary Table S11. Regression models assessing for interaction between baseline variables and treatment allocation on myocardial ECV at 52 weeks. 18

Supplementary Table S12. Multivariable regression model assessing for interaction between baseline variables and treatment allocation on myocardial ECV at 52 weeks. 19

Supplementary Figure S1. Interaction plots for baseline variables that modify treatment effect of pirfenidone on myocardial ECV at 52 weeks. 20

SUPPLEMENTARY TABLES

Supplementary Table S1. Association between patient characteristics and myocardial extracellular volume (ECV) at baseline

Baseline Covariate	Univariable Model				Multivariable Model		
	N	β-coefficient (SE)	95% CI	P-value	β-coefficient (SE)	95% CI	P-value
Age – yrs	107	0.04 (0.04)	-0.04 to 0.12	0.32			
Gender (Female vs Male)	107	-1.13 (0.60)	-2.32 to 0.05	0.06			
Hypertension (Yes vs No)	107	0.38 (0.81)	-1.23 to 1.99	0.64			
Diabetes (Yes vs No)	107	0.08 (0.68)	-1.26 to 1.42	0.91			
Atrial fibrillation (Yes vs No)	107	1.67 (0.59)	0.50 to 2.83	0.006			
Current or ex-smoker (Yes vs No)	107	-0.71 (0.62)	-1.94 to 0.52	0.26			
NYHA Class (II vs I)	107	-0.50 (1.34)	-3.16 to 2.16	0.71			
NYHA Class (III vs I)	107	0.46 (1.36)	-2.23 to 3.14	0.74			
Systolic blood pressure – mmHg	107	-0.04 (0.01)	-0.07 to -0.02	<0.001			
Diastolic blood pressure - mmHg	107	-0.07 (0.02)	-0.11 to -0.03	<0.001			
BMI - kg/m^2	107	-0.18 (0.05)	-0.28 to -0.08	<0.001	-0.19 (0.05)	-0.29 to -0.10	<0.001
eGFR - ml/min	107	-0.00 (0.02)	-0.04 to 0.03	0.86			
Haemoglobin - g/dL	106	-0.49 (0.20)	-0.89 to -0.09	0.02	-0.69 (0.18)	-1.05 to -0.34	<0.001
Log-NT-proBNP - pg/ml	107	1.39 (0.29)	0.82 to 1.97	<0.001	0.62 (0.36)	-0.09 to 1.33	0.09
HS Troponin T - pg/ml	107	0.03 (0.01)	0.01 to 0.05	0.003			
QRS duration – ms	107	-0.00 (0.02)	-0.03 to 0.03	0.96			
Infarct LGE (Yes vs No)	107	1.31 (0.66)	0.01 to 2.61	0.05			
LV end diastolic volume index - ml/m^2	107	-0.01 (0.02)	-0.04 to 0.03	0.64			
LV ejection fraction - %	107	-0.02 (0.03)	-0.09 to 0.05	0.57			
LV mass index - g/m^2	107	0.03 (0.02)	-0.01 to 0.07	0.20			
Average e’ - cm/s	107	0.12 (0.12)	-0.13 to 0.37	0.33			
Average E/e’ - cm/s	106	-0.01 (0.09)	-0.19 to 0.16	0.88			
Global longitudinal strain - %	106	0.03 (0.09)	-0.14 to 0.20	0.72			

Torsion - degrees/cm	105	-0.01 (0.47)	-0.94 to 0.92	0.99			
RV end diastolic volume index - ml/m^2	107	0.01 (0.02)	-0.03 to 0.04	0.75			
RV ejection fraction - %	107	-0.03 (0.03)	-0.09 to 0.03	0.35			
Pulmonary artery systolic pressure - mmHg	62	0.01 (0.03)	-0.05 to 0.08	0.67			
LA volume index - ml/m^2	107	0.05 (0.02)	0.02 to 0.08	0.003			
LA strain (reservoir) - %	105	-0.13 (0.04)	-0.20 to -0.06	<0.001	-0.11 (0.04)	-0.20 to -0.03	0.01
LA strain (booster) - %	59	-0.23 (0.08)	-0.39 to -0.06	0.008			
LA strain (conduit) - %	105	-0.05 (0.08)	-0.21 to 0.11	0.54			
Aortic distensibility - 10^{-3}/mmHg	107	0.43 (0.35)	-0.27 to 1.13	0.22	0.69 (0.29)	0.11 to 1.27	0.02
Pulse Wave Velocity - m/s	97	-0.01 (0.06)	-0.14 to 0.11	0.85			
6-minute walk test - m	107	-0.00 (0.00)	-0.01 to 0.00	0.54			
KCCQ Clinical Summary Score	105	0.01 (0.01)	-0.01 to 0.04	0.33			

Table S1 Legend. All patients who underwent baseline assessment and had complete covariate data were included in analyses. The multivariable model included patients with complete covariate data (n=104). Variables for which P-value <0.3 in univariable regression model included within stepwise forward selection multivariable regression model. BMI – body mass index; CI – confidence interval; eGFR – estimated glomerular filtration rate; HS-Troponin T – high-sensitivity troponin T; KCCQ – Kansas City Cardiomyopathy Questionnaire; LA – left atrial; LGE – late gadolinium enhancement; LV – left ventricular; NT-proBNP – N-terminal pro B-type natriuretic peptide; NYHA – New York Heart Association; RV – right ventricular; SE – standard error.

Supplementary Table S2. Association between patient characteristics and absolute myocardial extracellular matrix (ECM) volume at baseline

Baseline Covariate	Univariable Model				Multivariable Model		
	N	β-coefficient (SE)	95% CI	P-value	β-coefficient (SE)	95% CI	P-value
Age – yrs	107	-0.42 (0.14)	-0.70 to -0.15	0.003			
Gender (Female vs Male)	107	-12.78 (1.78)	-16.31 to -9.25	<0.001	-11.91 (1.49)	-14.87 to -8.94	<0.001
Hypertension (Yes vs No)	107	0.85 (2.90)	-4.91 to 6.60	0.77			
Diabetes (Yes vs No)	107	1.95 (2.41)	-2.83 to 6.73	0.42			
Atrial fibrillation (Yes vs No)	107	0.64 (2.18)	-3.69 to 4.97	0.76			
Current or ex-smoker (Yes vs No)	107	1.56 (2.23)	-2.86 to 5.98	0.49			
NYHA Class (II vs I)	107	-6.75 (4.80)	-16.28 to 2.78	0.16			
NYHA Class (III vs I)	107	-5.83 (4.86)	-15.47 to 3.81	0.23			
Systolic blood pressure – mmHg	107	0.03 (0.05)	-0.07 to 0.12	0.60			
Diastolic blood pressure - mmHg	107	0.02 (0.07)	-0.13 to 0.16	0.83			
BMI - kg/m^2	107	0.12 (0.20)	-0.27 to 0.50	0.56			
eGFR - ml/min	107	0.13 (0.06)	0.01 to 0.26	0.04			
Haemoglobin - g/dL	106	0.22 (0.75)	-1.27 to 1.70	0.77			
Log-NT-proBNP - pg/ml	107	1.72 (1.14)	-0.54 to 3.98	0.13			
HS Troponin T - pg/ml	107	0.07 (0.04)	-0.00 to 0.14	0.05			
QRS duration – ms	107	0.12 (0.05)	0.01 to 0.22	0.04			
Infarct LGE (Yes vs No)	107	3.62 (2.37)	-1.07, 8.32	0.13	5.53 (1.63)	2.29 to 8.77	0.001
LV end diastolic volume index - ml/m^2	107	0.32 (0.05)	0.22 to 0.43	<0.001	0.30 (0.05)	0.20 to 0.41	<0.001
LV ejection fraction - %	107	-0.36 (0.12)	-0.60 to -0.13	0.003	0.26 (0.10)	0.07 to 0.46	0.008
LV mass index - g/m^2	107	0.66 (0.03)	0.59 to 0.72	<0.001			
Average e' - cm/s	107	0.46 (0.45)	-0.42 to 1.35	0.30			
Average E/e' - cm/s	106	0.17 (0.32)	-0.46 to 0.81	0.59			
Global longitudinal strain - %	106	0.66 (0.30)	0.07 to 1.25	0.03			
Torsion - degrees/cm	105	-2.78 (1.64)	-6.02 to 0.46	0.09	-2.28 (1.13)	-4.53 to -0.03	0.05

RV end diastolic volume index - ml/m^2	107	0.34 (0.06)	0.23 to 0.46	<0.001	0.13 (0.06)	0.02 to 0.24	0.02
RV ejection fraction - %	107	-0.13 (0.11)	-0.36 to 0.09	0.24			
Pulmonary artery systolic pressure - mmHg	62	-0.04 (0.12)	-0.27 to 0.19	0.73			
LA volume index - ml/m^2	107	0.18 (0.06)	0.06 to 0.29	0.003			
LA strain (reservoir) - %	105	-0.23 (0.14)	-0.50 to 0.05	0.10			
LA strain (booster) - %	59	-0.48 (0.34)	-1.16 to 0.20	0.16			
LA strain (conduit) - %	105	-0.48 (0.28)	-1.04 to 0.09	0.10	-0.41 (0.18)	-0.77 to -0.05	0.03
Aortic distensibility - 10^{-3}/mmHg	107	0.68 (1.27)	-1.83 to 3.19	0.59			
Pulse Wave Velocity - m/s	97	-0.23 (0.23)	-0.69 to 0.23	0.32			
6-minute walk test - m	107	0.01 (0.01)	-0.00 to 0.03	0.13			
KCCQ Clinical Summary Score	105	0.04 (0.05)	-0.07 to 0.15	0.46			

Table S2 Legend. All patients who underwent baseline assessment and had complete covariate data were included in analyses. The multivariable model included patients with complete covariate data (n=103). Variables for which P-value <0.3 in univariable regression model included within stepwise forward selection multivariable regression model. BMI – body mass index; CI – confidence interval; eGFR – estimated glomerular filtration rate; HS-Troponin T – high-sensitivity troponin T; KCCQ – Kansas City Cardiomyopathy Questionnaire; LA – left atrial; LGE – late gadolinium enhancement; LV – left ventricular; NT-proBNP – N-terminal pro B-type natriuretic peptide; NYHA – New York Heart Association; RV – right ventricular; SE – standard error.

Supplementary Table S3. Association between patient characteristics and absolute myocardial extracellular matrix (ECM) volume at baseline (clinical model)

Baseline covariate	Estimate (SE)	95% CI	P-value
Age – yrs	-0.30 (0.12)	-0.54 to -0.06	0.02
Gender (Female vs Male)	-11.78 (1.74)	-15.23 to -8.33	<0.001
BMI – kg/m^2	0.25 (0.15)	-0.05 to 0.55	0.10
Diabetes (Yes vs No)	-1.22 (1.85)	-4.90 to 2.56	0.51
Atrial fibrillation (Yes vs No)	-6.49 (2.58)	-11.61 to -1.38	0.01
Log NT-proBNP – pg/ml	1.95 (1.15)	-0.33 to 4.24	0.09
LA strain (reservoir) - %	-0.38 (0.17)	-0.72 to -0.04	0.03
Systolic blood pressure – mmHg	0.06 (0.04)	-0.02 to 0.13	0.13
RV end-diastolic volume index – ml/m^2	0.22 (0.05)	0.12 to 0.33	<0.001
Global longitudinal strain - %	0.05 (0.26)	-0.47 to 0.56	0.85

Table S3 Legend. All patients who underwent baseline assessment and had complete covariate data were included in the analyses (n=104). Clinical multivariable model with selected baseline covariates. BMI – body mass index; CI – confidence interval; LA – left atrial; NT-proBNP – N-terminal pro B-type natriuretic peptide; RV – right ventricular; SE – standard error.

Supplementary Table S4. Association between patient characteristics and absolute myocardial cell volume at baseline.

Baseline Covariate	Univariable Model				Multivariable Model		
	N	β-coefficient (SE)	95% CI	P-value	β-coefficient (SE)	95% CI	P-value
Age – yrs	107	-1.12 (0.30)	-1.71 to -0.53	<0.001			
Gender (Female vs Male)	107	-25.73 (4.07)	-33.80 to -17.67	<0.001	-27.19 (2.76)	-32.67 to -21.71	<0.001
Hypertension (Yes vs No)	107	-0.83 (6.38)	-13.49 to 11.83	0.90			
Diabetes (Yes vs No)	107	3.84 (5.30)	-6.68 to 14.36	0.47			
Atrial fibrillation (Yes vs No)	107	-4.96 (4.78)	-14.44 to 4.51	0.30			
Current or ex-smoker (Yes vs No)	107	6.83 (4.87)	-2.82 to 16.48	0.16			
NYHA Class (II vs I)	107	-11.37 (10.59)	-32.36 to 9.63	0.29			
NYHA Class (III vs I)	107	-13.19 (10.71)	-34.43 to 8.05	0.22			
Systolic blood pressure – mmHg	107	0.22 (0.10)	0.01 to 0.42	0.04	0.21 (0.06)	0.10 to 0.32	<0.001
Diastolic blood pressure - mmHg	107	0.31 (0.16)	-0.00 to 0.63	0.05			
BMI - kg/m^2	107	1.07 (0.42)	0.25 to 1.90	0.01	1.43 (0.23)	0.97 to 1.89	<0.001
eGFR - ml/min	107	0.35 (0.14)	0.07 to 0.63	0.01			
Haemoglobin - g/dL	106	2.51 (1.60)	-0.67 to 5.69	0.12			
Log-NT-proBNP - pg/ml	107	-1.51 (2.53)	-6.52 to 3.51	0.55			
HS Troponin T - pg/ml	107	0.04 (0.08)	-0.12 to 0.20	0.63			
QRS duration – ms	107	0.30 (0.12)	0.06 to 0.54	0.01	0.15 (0.07)	0.02 to 0.28	0.03
Infarct LGE (Yes vs No)	107	2.79 (5.26)	-7.63 to 13.22	0.60			
LV end diastolic volume index - ml/m^2	107	0.79 (0.11)	0.57 to 1.01	<0.001	0.76 (0.08)	0.61 to 0.92	<0.001
LV ejection fraction - %	107	-0.75 (0.27)	-1.28 to -0.23	0.005	0.52 (0.17)	0.17 to 0.86	0.004
LV mass index - g/m^2	107	1.42 (0.08)	1.27 to 1.57	<0.001			
Average e' - cm/s	107	0.60 (0.98)	-1.36 to 2.55	0.55			
Average E/e' - cm/s	106	0.46 (0.70)	-0.93 to 1.86	0.51			
Global longitudinal strain - %	106	1.58 (0.65)	0.29 to 2.87	0.02	1.41 (0.39)	0.64 to 2.17	<0.001
Torsion - degrees/cm	105	-6.47 (3.60)	-13.61 to 0.66	0.08			
RV end diastolic volume index - ml/m^2	107	0.79 (0.13)	0.53 to 1.04	<0.001			

RV ejection fraction - %	107	-0.28 (0.25)	-0.77 to 0.22	0.27			
Pulmonary artery systolic pressure - mmHg	62	-0.17 (0.23)	-0.64 to 0.30	0.47			
LA volume index - ml/m^2	107	0.21 (0.13)	-0.05 to 0.47	0.11			
LA strain (reservoir) - %	105	-0.02 (0.30)	-0.62 to 0.59	0.95			
LA strain (booster) - %	59	-0.13 (0.75)	-1.63 to 1.37	0.86			
LA strain (conduit) - %	105	-0.96 (0.63)	-2.20 to 0.28	0.13			
Aortic distensibility - 10^{-3}/mmHg	107	0.06 (2.79)	-5.47 to 5.59	0.98			
Pulse Wave Velocity - m/s	97	-0.56 (0.51)	-1.58 to 0.45	0.27			
6-minute walk test - m	107	0.04 (0.02)	-0.00 to 0.08	0.06			
KCCQ Clinical Summary Score	105	0.04 (0.12)	-0.19 to 0.27	0.72			

Table S4 Legend. All patients who underwent baseline assessment and had complete covariate data were included in analyses. The multivariable model included patients with complete covariate data (n=106). Variables for which P-value <0.3 in univariable regression model included within stepwise forward selection multivariable regression model. BMI – body mass index; CI – confidence interval; eGFR – estimated glomerular filtration rate; HS-Troponin T – high-sensitivity troponin T; KCCQ – Kansas City Cardiomyopathy Questionnaire; LA – left atrial; LGE – late gadolinium enhancement; LV – left ventricular; NT-proBNP – N-terminal pro B-type natriuretic peptide; NYHA – New York Heart Association; RV – right ventricular; SE – standard error.

Supplementary Table S5. Association between patient characteristics and absolute myocardial cell volume at baseline (clinical model)

Baseline covariate	Estimate (SE)	95% CI	P-value
Age – yrs	-0.48 (0.24)	-0.96 to 0.01	0.05
Gender (Female vs Male)	-26.75 (3.46)	-33.62 to -19.88	<0.001
BMI – kg/m^2	1.31 (0.30)	0.71 to 1.92	<0.001
Diabetes (Yes vs No)	-3.50 (3.69)	-10.83 to 3.83	0.34
Atrial fibrillation (Yes vs No)	-13.47 (5.13)	-23.67 to -3.28	0.01
Log NT-proBNP – pg/ml	1.17 (2.29)	-3.38 to 5.73	0.61
LA strain (reservoir) - %	-0.43 (0.34)	-1.10 to 0.25	0.21
Systolic blood pressure – mmHg	0.22 (0.07)	0.08 to 0.37	0.004
RV end-diastolic volume index – ml/m^2	0.46 (0.11)	0.25 to 0.67	<0.001
Global longitudinal strain - %	0.99 (0.52)	-0.04 to 2.02	0.06

Table S5 Legend. All patients who underwent baseline assessment and had complete covariate data were included in analyses (n=104). Clinical multivariable model with selected baseline covariates. BMI – body mass index; CI – confidence interval; LA – left atrial; NT-proBNP – N-terminal pro B-type natriuretic peptide; RV – right ventricular; SE – standard error.

Supplementary Table S6. Associations between baseline characteristics and change in myocardial extracellular volume (ECV) from baseline to 52 weeks

Baseline Covariate	Univariable Model				Multivariable Model		
	N	β-coefficient (SE)	95% CI	P-value	β-coefficient (SE)	95% CI	P-value
Age – yrs	80	-0.00 (0.03)	-0.07 to 0.06	0.96			
Gender (Female vs Male)	80	-0.68 (0.44)	-1.56 to 0.20	0.13			
Hypertension (Yes vs No)	80	0.06 (0.61)	-1.15 to 1.27	0.93			
Diabetes (Yes vs No)	80	0.85 (0.51)	-0.17 to 1.87	0.10			
Atrial fibrillation (Yes vs No)	80	0.51 (0.45)	-0.38 to 1.40	0.25			
Current or ex-smoker (Yes vs No)	80	0.73 (0.47)	-0.21 to 1.67	0.14			
NYHA Class (II vs I)	80	1.01 (0.98)	-0.94 to 2.95	0.31			
NYHA Class (III vs I)	80	0.36 (0.97)	-1.57 to 2.30	0.71			
Systolic blood pressure – mmHg	80	-0.01 (0.01)	-0.03 to 0.01	0.49			
Diastolic blood pressure - mmHg	80	0.02 (0.02)	-0.01 to 0.05	0.21			
BMI - kg/m^2	80	0.02 (0.04)	-0.06 to 0.10	0.59			
eGFR - ml/min	80	0.00 (0.01)	-0.02 to 0.03	0.79			
Haemoglobin - g/dL	80	0.06 (0.16)	-0.25 to 0.37	0.71			
Log-NT-proBNP - pg/ml	80	0.38 (0.25)	-0.10 to 0.87	0.12			
HS Troponin T - pg/ml	80	0.03 (0.01)	-0.00 to 0.05	0.08			
QRS duration – ms	80	-0.03 (0.01)	-0.05 to -0.00	0.04	-0.03 (0.01)	-0.05 to -0.00	0.02
Infarct LGE (Yes vs No)	80	1.20 (0.45)	0.30 to 2.10	0.009	1.13 (0.43)	0.27 to 2.00	0.01
LV end diastolic volume index - ml/m^2	80	-0.01 (0.01)	-0.03 to 0.02	0.59			
LV ejection fraction - %	80	-0.04 (0.03)	-0.09 to 0.02	0.18			
LV mass index - g/m^2	80	0.03 (0.01)	-0.00 to 0.05	0.07	0.03 (0.01)	0.00 to 0.05	0.05
Average e' - cm/s	80	0.04 (0.09)	-0.14 to 0.22	0.64			
Average E/e' - cm/s	79	0.05 (0.07)	-0.08 to 0.18	0.47			
Global longitudinal strain - %	80	0.14 (0.06)	0.02 to 0.26	0.02			
Torsion - degrees/cm	78	-0.21 (0.34)	-0.88 to 0.46	0.53			
RV end diastolic volume index - ml/m^2	80	-0.00 (0.01)	-0.03 to 0.03	0.84			
RV ejection fraction - %	80	-0.02 (0.02)	-0.07 to 0.03	0.39			
Pulmonary artery systolic pressure - mmHg	53	-0.01 (0.02)	-0.05 to 0.03	0.63			

LA volume index - ml/m^2	80	0.01 (0.01)	-0.01 to 0.04	0.22			
LA strain (reservoir) - %	79	-0.05 (0.03)	-0.11 to 0.01	0.10			
LA strain (booster) - %	41	-0.07 (0.07)	-0.22 to 0.08	0.33			
LA strain (conduit) - %	79	-0.06 (0.06)	-0.18 to 0.07	0.36			
Aortic distensibility - 10^{-3}/mmHg	80	0.00 (0.27)	-0.54 to 0.54	0.99			
Pulse Wave Velocity - m/s	72	0.06 (0.05)	-0.03 to 0.15	0.23			
6-minute walk test - m	80	0.00 (0.00)	-0.00 to 0.00	0.63			
KCCQ Clinical Summary Score	79	0.01 (0.01)	-0.01 to 0.03	0.43			

Table S6 Legend. All patients who underwent baseline assessment and had complete covariate data were included in analyses. The multivariable model included patients with complete covariate data (n=80). Variables for which P-value <0.3 in univariable regression model included within stepwise forward selection multivariable regression model, adjusted for treatment allocation. BMI – body mass index; CI – confidence interval; eGFR – estimated glomerular filtration rate; HS-Troponin T – high-sensitivity troponin T; KCCQ – Kansas City Cardiomyopathy Questionnaire; LA – left atrial; LGE – late gadolinium enhancement; LV – left ventricular; NT-proBNP – N-terminal pro B-type natriuretic peptide; NYHA – New York Heart Association; RV – right ventricular; SE – standard error.

Supplementary Table S7. Association between patient characteristics and change in absolute myocardial extracellular matrix (ECM) volume from baseline to 52 weeks

Baseline Covariate	Univariable Model				Multivariable Model		
	N	β-coefficient (SE)	95% CI	P-value	β-coefficient (SE)	95% CI	P-value
Age – yrs	80	-0.05 (0.07)	-0.19 to 0.09	0.47			
Gender (Female vs Male)	80	0.41 (0.93)	-1.45 to 2.27	0.66			
Hypertension (Yes vs No)	80	-1.04 (1.26)	-3.55 to 1.48	0.41			
Diabetes (Yes vs No)	80	2.22 (1.06)	0.12 to 4.33	0.04	2.22 (1.06)	0.12 to 4.33	0.04
Atrial fibrillation (Yes vs No)	80	1.24 (0.93)	-0.61 to 3.09	0.19			
Current or ex-smoker (Yes vs No)	80	-0.49 (1.00)	-2.48 to 1.49	0.62			
NYHA Class (II vs I)	80	-0.88 (2.06)	-4.98 to 3.21	0.67			
NYHA Class (III vs I)	80	-1.83 (2.04)	-5.90 to 2.24	0.37			
Systolic blood pressure – mmHg	80	-0.03 (0.02)	-0.07 to 0.02	0.22			
Diastolic blood pressure - mmHg	80	-0.06 (0.03)	-0.12 to 0.01	0.09			
BMI - kg/m^2	80	0.03 (0.09)	-0.14 to 0.20	0.73			
eGFR - ml/min	80	-0.07 (0.03)	-0.12 to -0.01	0.02			
Haemoglobin - g/dL	80	-0.56 (0.32)	-1.20 to 0.08	0.09			
Log-NT-proBNP - pg/ml	80	0.48 (0.52)	-0.55 to 1.51	0.35			
HS Troponin T - pg/ml	80	0.04 (0.03)	-0.02 to 0.10	0.21			
QRS duration – ms	80	-0.02 (0.03)	-0.07 to 0.03	0.44			
Infarct LGE (Yes vs No)	80	2.24 (0.95)	0.35 to 4.14	0.02			
LV end diastolic volume index - ml/m^2	80	-0.06 (0.02)	-0.10 to -0.01	0.02			
LV ejection fraction - %	80	0.01 (0.06)	-0.10 to 0.12	0.89			
LV mass index - g/m^2	80	-0.05 (0.03)	-0.11 to 0.01	0.10			
Average e' - cm/s	80	0.26 (0.19)	-0.11 to 0.63	0.17			
Average E/e' - cm/s	79	-0.12 (0.14)	-0.39 to 0.15	0.38			
Global longitudinal strain - %	80	0.10 (0.13)	-0.16 to 0.36	0.46			
Torsion - degrees/cm	78	0.41 (0.73)	-1.04 to 1.86	0.58			
RV end diastolic volume index - ml/m^2	80	-0.03 (0.03)	-0.09 to 0.03	0.30			

RV ejection fraction - %	80	-0.01 (0.05)	-0.11 to 0.09	0.88			
Pulmonary artery systolic pressure - mmHg	53	-0.02 (0.04)	-0.11 to 0.07	0.64			
LA volume index - ml/m^2	80	0.01 (0.02)	-0.03 to 0.06	0.55			
LA strain (reservoir) - %	79	-0.12 (0.06)	-0.24 to 0.00	0.06			
LA strain (booster) - %	41	-0.25 (0.18)	-0.61 to 0.12	0.18			
LA strain (conduit) - %	79	-0.08 (0.13)	-0.34 to 0.18	0.52			
Aortic distensibility - 10^{-3}/mmHg	80	-0.19 (0.56)	-1.31 to 0.93	0.74			
Pulse Wave Velocity - m/s	72	-0.14 (0.10)	-0.33 to 0.06	0.17			
6-minute walk test - m	80	0.00 (0.00)	-0.01 to 0.01	0.54			
KCCQ Clinical Summary Score	79	0.01 (0.02)	-0.04 to 0.06	0.64			

Table S7 Legend. All patients who underwent baseline assessment and had complete covariate data were included in analyses. The multivariable model included patients with complete covariate data (n=80). Variables for which P-value <0.3 in univariable regression model included within stepwise forward selection multivariable regression model, adjusted for treatment allocation. BMI – body mass index; CI – confidence interval; eGFR – estimated glomerular filtration rate; HS-Troponin T – high-sensitivity troponin T; KCCQ – Kansas City Cardiomyopathy Questionnaire; LA – left atrial; LGE – late gadolinium enhancement; LV – left ventricular; NT-proBNP – N-terminal pro B-type natriuretic peptide; NYHA – New York Heart Association; RV – right ventricular; SE – standard error.

Supplementary Table S8. Association between patient characteristics and change in absolute myocardial extracellular matrix (ECM) volume from baseline to 52 weeks (clinical model)

Baseline covariate	Estimate (SE)	95% CI	P-value
Allocation (Pirfenidone vs Placebo)	-2.85 (1.02)	-4.87 to -0.82	0.007
Age – yrs	-0.06 (0.08)	-0.22 to 0.11	0.49
Gender (Female vs Male)	0.89 (0.99)	-1.09 to 2.87	0.37
Diabetes (Yes vs No)	2.14 (1.23)	-0.32 to 4.60	0.09
Log NT-proBNP – pg/ml	-0.02 (0.69)	-1.40 to 1.35	0.98
LA strain (reservoir) - %	-0.12 (0.09)	-0.29 to 0.05	0.17
Systolic blood pressure – mmHg	-0.01 (0.02)	-0.06 to 0.04	0.74
RV end-diastolic volume index – ml/m^2	-0.02 (0.03)	-0.08 to 0.04	0.51
Global longitudinal strain - %	-0.01 (0.17)	-0.35 to 0.34	0.96

Table S8 Legend. All patients who completed the study and had complete covariate data were included in analyses (n=79). Clinical multivariable model with selected baseline covariates. CI – confidence interval; LA – left atrial; NT-proBNP – N-terminal pro B-type natriuretic peptide; RV – right ventricular; SE – standard error.

Supplementary Table S9. Association between patient characteristics and change in absolute myocardial cell volume from baseline to 52 weeks

Baseline Covariate	Univariable Model				Multivariable Model		
	N	β-coefficient (SE)	95% CI	P-value	β-coefficient (SE)	95% CI	P-value
Age – yrs	80	-0.10 (0.16)	-0.41 to 0.22	0.54			
Gender (Female vs Male)	80	3.29 (2.11)	-0.90 to 7.49	0.12			
Hypertension (Yes vs No)	80	-2.38 (2.89)	-8.13 to 3.37	0.41			
Diabetes (Yes vs No)	80	2.05 (2.48)	-2.89 to 6.99	0.41			
Atrial fibrillation (Yes vs No)	80	1.06 (2.15)	-3.22 to 5.33	0.62			
Current or ex-smoker (Yes vs No)	80	-3.25 (2.25)	-7.74 to 1.24	0.15			
NYHA Class (II vs I)	80	-4.33 (4.72)	-13.73 to 5.08	0.36			
NYHA Class (III vs I)	80	-4.10 (4.69)	-13.44 to 5.24	0.38			
Systolic blood pressure – mmHg	80	-0.05 (0.05)	-0.14 to 0.05	0.34			
Diastolic blood pressure - mmHg	80	-0.22 (0.07)	-0.36 to -0.08	0.003	-0.21 (0.07)	-0.35 to -0.08	0.003
BMI - kg/m^2	80	-0.12 (0.20)	-0.51 to 0.27	0.54			
eGFR - ml/min	80	-0.17 (0.06)	-0.29 to -0.04	0.009	-0.16 (0.06)	-0.28 to -0.04	0.008
Haemoglobin - g/dL	80	-1.55 (0.73)	-3.01 to -0.10	0.04			
Log-NT-proBNP - pg/ml	80	-0.15 (1.19)	-2.52 to 2.22	0.90			
HS Troponin T - pg/ml	80	0.00 (0.07)	-0.14 to 0.14	0.99			
QRS duration – ms	80	0.05 (0.06)	-0.07 to 0.17	0.41			
Infarct LGE (Yes vs No)	80	1.37 (2.25)	-3.11 to 5.85	0.55			
LV end diastolic volume index - ml/m^2	80	-0.14 (0.05)	-0.24 to -0.03	0.01			
LV ejection fraction - %	80	0.15 (0.12)	-0.10 to 0.40	0.23			
LV mass index - g/m^2	80	-0.24 (0.06)	-0.37 to -0.11	<0.001			
Average e' - cm/s	80	0.36 (0.43)	-0.50 to 1.21	0.41			
Average E/e' - cm/s	79	-0.52 (0.31)	-1.14 to 0.09	0.09			
Global longitudinal strain - %	80	-0.25 (0.30)	-0.84 to 0.34	0.40			
Torsion - degrees/cm	78	1.81 (1.63)	-1.44 to 5.06	0.27			
RV end diastolic volume index - ml/m^2	80	-0.09 (0.07)	-0.22 to 0.04	0.16			
RV ejection fraction - %	80	0.08 (0.12)	-0.16 to 0.31	0.51			
Pulmonary artery systolic pressure - mmHg	53	-0.01 (0.09)	-0.20 to 0.18	0.89			

LA volume index - ml/m^2	80	-0.04 (0.06)	-0.15 to 0.07	0.49			
LA strain (reservoir) - %	79	-0.09 (0.14)	-0.38 to 0.19	0.53			
LA strain (booster) - %	41	-0.24 (0.38)	-1.01 to 0.54	0.54			
LA strain (conduit) - %	79	-0.01 (0.30)	-0.61 to 0.59	0.98			
Aortic distensibility - 10^{-3}/mmHg	80	-0.34 (1.29)	-2.90 to 2.22	0.79			
Pulse Wave Velocity - m/s	72	-0.46 (0.23)	-0.92 to 0.01	0.05			
6-minute walk test - m	80	0.00 (0.01)	-0.01 to 0.02	0.67			
KCCQ Clinical Summary Score	79	-0.00 (0.05)	-0.11 to 0.10	0.97			

Table S9 Legend. All patients who underwent baseline assessment and had complete covariate data were included in analyses. The multivariable model included patients with complete covariate data (n=80). Variables for which P-value <0.3 in univariable regression model included within stepwise forward selection multivariable regression model. BMI – body mass index; CI – confidence interval; eGFR – estimated glomerular filtration rate; HS-Troponin T – high-sensitivity troponin T; KCCQ – Kansas City Cardiomyopathy Questionnaire; LA – left atrial; LGE – late gadolinium enhancement; LV – left ventricular; NT-proBNP – N-terminal pro B-type natriuretic peptide; NYHA – New York Heart Association; RV – right ventricular; SE – standard error.

Supplementary Table S10. Association between patient characteristics and change in absolute myocardial cell volume from baseline to 52 weeks (clinical model)

Baseline covariate	Estimate (SE)	95% CI	P-value
Allocation (Pirfenidone vs Placebo)	-2.77 (2.36)	-7.49 to 1.95	0.25
Age – yrs	-0.21 (0.19)	-0.59 to 0.17	0.28
Gender (Female vs Male)	2.84 (2.30)	-1.76 to 7.43)	0.22
Diabetes (Yes vs No)	1.25 (2.87)	-4.47 to 6.96	0.66
Log NT-proBNP – pg/ml	-0.38 (1.60)	-3.58 to 2.82	0.81
LA strain (reservoir) - %	-0.24 (0.20)	-0.64 to 0.16	0.23
Systolic blood pressure – mmHg	-0.02 (0.05)	-0.13 to 0.08	0.65
RV end-diastolic volume index – ml/m^2	-0.08 (0.07)	-0.22 to 0.07	0.31
Global longitudinal strain - %	-0.57 (0.40)	-1.38 to 0.24	0.16

Table S10 Legend. All patients who completed the study and had complete covariate data were included in analyses (n=79). Clinical multivariable model with selected baseline covariates. CI – confidence interval; LA – left atrial; NT-proBNP – N-terminal pro B-type natriuretic peptide; RV – right ventricular; SE – standard error.

Supplementary Table S11. Regression models assessing for interaction between baseline variables and treatment allocation on myocardial ECV at 52 weeks.

Baseline Covariate	P-value
Age – yrs	0.25
Gender (Female vs Male)	0.63
Hypertension (Yes vs No)	0.21
Diabetes (Yes vs No)	0.49
Atrial fibrillation (Yes vs No)	0.32
Current or ex-smoker (Yes vs No)	0.54
NYHA Class (II vs I)	0.10
Systolic blood pressure – mmHg	0.99
Diastolic blood pressure - mmHg	0.90
BMI - kg/m^2	0.25
eGFR - ml/min	0.72
Haemoglobin - g/dL	0.10
Log-NT-proBNP - pg/ml	0.19
HS Troponin T - pg/ml	0.36
QRS duration – ms	0.007
Infarct LGE (Yes vs No)	0.047
LV end diastolic volume index - ml/m^2	0.08
LV ejection fraction - %	0.57
LV mass index - g/m^2	0.45
Average e’ - cm/s	0.36
Average E/e’ - cm/s	0.71
Global longitudinal strain - %	0.035
Torsion - degrees/cm	0.58
RV end diastolic volume index - ml/m^2	0.20
RV ejection fraction - %	0.74
Pulmonary artery systolic pressure - mmHg	0.45
LA volume index - ml/m^2	0.70
LA strain (reservoir) - %	0.13
LA strain (booster) - %	0.38
LA strain (conduit) - %	0.021
Aortic distensibility - 10^{-3}/mmHg	0.66
Pulse Wave Velocity - m/s	0.34
6-minute walk test - m	0.72
KCCQ Clinical Summary Score	0.27

Table S11 Legend. Ordinary least squares regression was used to model myocardial ECV at 52 weeks (adjusted for baseline ECV), including an interaction term between treatment allocation and each baseline covariate. P-values from the interaction term for each baseline variable are presented. BMI – body mass index; eGFR – estimated glomerular filtration rate; HS-Troponin T – high-sensitivity troponin T; KCCQ – Kansas City Cardiomyopathy Questionnaire; LA – left atrial; LGE – late gadolinium enhancement; LV – left ventricular; NT-proBNP – N-terminal pro B-type natriuretic peptide; NYHA – New York Heart Association; RV – right ventricular; SE – standard error.

Supplementary Table S12. Multivariable regression model assessing for interaction between baseline variables and treatment allocation on myocardial ECV at 52 weeks.

Variable	Estimate (SE)	95% CI	P-value
Allocation (Pirfenidone vs Placebo)	-9.74 (2.65)	-15.01 to -4.46	<0.001
Baseline myocardial ECV (%)	0.99 (0.08)	0.83 to 1.14	<0.001
QRS duration (ms)	-0.05 (0.02)	-0.09 to -0.02	0.003
LA strain (conduit) (%)	-0.22 (0.10)	-0.43 to -0.02	0.03
Interaction between QRS and Allocation	0.06 (0.02)	0.01 to 0.11	0.02
Interaction between LA strain and Allocation	0.26 (0.13)	0.01 to 0.52	0.04

Table S12 Legend. Interaction terms for the two most significant interactions (QRS duration and LA strain [conduit]) remained significant in the above multivariable model, however addition of the third most significant interaction (global longitudinal strain) rendered the existing interactions non-significant. CI – confidence interval; ECV – extracellular matrix volume; LA – left atrial; SE – standard error.

Supplementary Figure S1. Interaction plots for baseline variables that modify treatment effect of pirfenidone on myocardial ECV at 52 weeks.

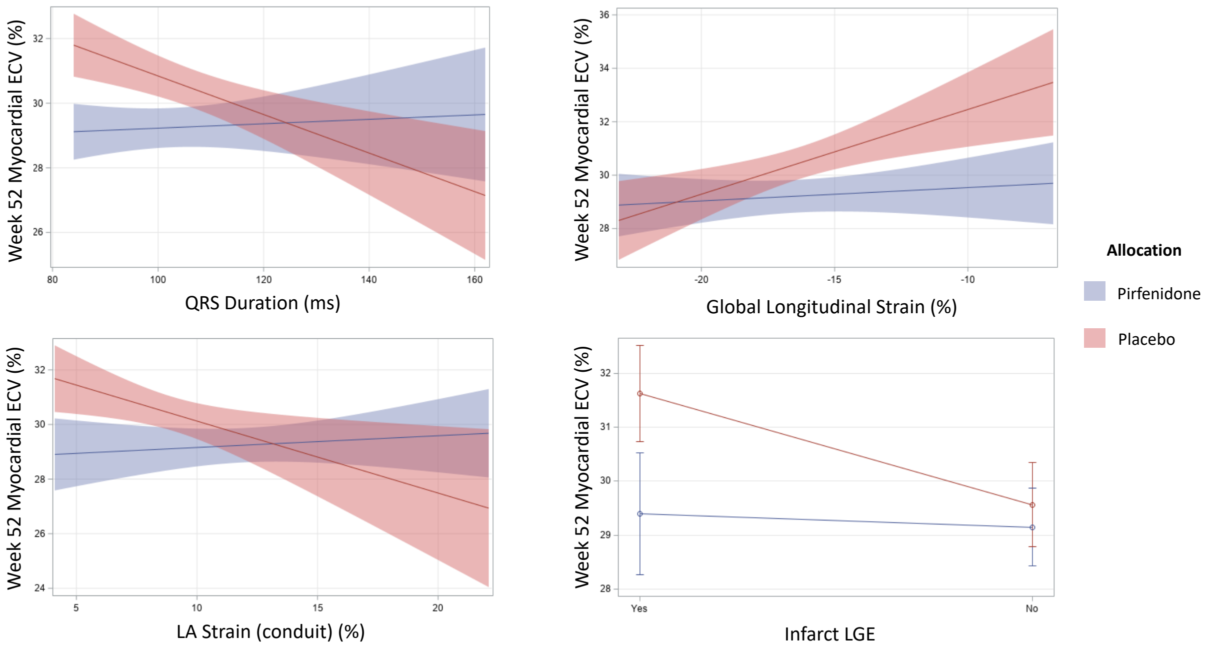

Figure S1 Legend. Interaction plots demonstrating the direction of effect associated with each variable with 95% confidence intervals. Individual interaction models demonstrated that a shorter QRS duration, impaired global longitudinal strain, impaired left atrial strain (conduit), and the presence of an infarct were associated with greater treatment effect of pirfenidone on ECV at 52 weeks (adjusted for baseline ECV). No baseline variable independently modified the treatment effect of pirfenidone on multivariable modelling. ECV – extracellular matrix volume; LA – left atrial; LGE – late gadolinium enhancement.
